# Financial sector and economic growth amid external uncertainty shocks: Insights into emerging economies

**DOI:** 10.1371/journal.pone.0259303

**Published:** 2021-11-11

**Authors:** Emmanuel Asafo-Adjei, Ebenezer Boateng, Zangina Isshaq, Anthony Adu-Asare Idun, Peterson Owusu Junior, Anokye M. Adam

**Affiliations:** Department of Finance, School of Business, University of Cape Coast, Cape Coast, Ghana; University of Almeria, SPAIN

## Abstract

The study aims to shed new lights on the lead-lag relationships between the financial sector (RFSI) and economic growth (GDP) in the midst of global economic policy uncertainty (GEPU) shocks for BRICS economies. Hence, the bivariate, partial, and wavelet multiple correlations techniques are employed. From the bivariate analysis, we document positive bi-directional causality between the RFSI and economic growth over the sample period. The partial wavelet reveals that GEPU shocks distort the significance and directional comovements between the RFSI and GDP. Moreover, the outcome from the wavelet multiple cross correlations (WMCC) indicates that the RFSI is a first mover at most time scales for the BRICS economies. This is followed by GEPU which either leads or lags for most scales, especially for South Africa. The impact of GEPU on RFSI and GDP is worst for South Africa in about four cases in the medium-, and long-terms. This signifies that South Africa’s financial markets and economic growth are vulnerable to GEPU. However, the impetus for GEPU to drive the comovements between the financial sector and economic activity was less pronounced in the pre-COVID analysis conducted with the WMCC. The study supports both the supply-leading and demand-following hypotheses. Our findings also underscore the need for policymakers, investors and academics alike to incessantly observe the dynamics between finance and growth across time and periodicity while considering adverse shocks from global economic policy uncertainty in tandem.

## 1. Introduction

The global financial crisis (GFC) in 2007–2009 has brought to light the importance of an efficient financial sector to the global economy. Following a series of recessions witnessed by emerging markets and the GFC, it has become blatant to practitioners, regulators, and researchers that the financial sector and macroeconomic variables are interrelated. Meanwhile, sustained economic growth is the primary focus of every economy. Haldane [1] re-echoed this when he averred that sustaining economic growth is the single most important determinant of the living standards of the society. Due to this, a copious amount of academic literature from finance and economic scholars has arguably been devoted to exploring the determinants of economic growth [2–5]. In this regard, the finance-growth nexus has become one of the oldest debates in finance and economic literature.

Early economists sharply disagreed on how the financial sector affects growth and development. Bauer, Meier, and Seers [3] did not consider finance worthy of discussion in their series of essays on “pioneers in economic development”. Similarly, Lucas [6] on the “mechanics of economic development” dismissed finance as an exaggerated factor in the determinants of growth. In the extreme form, Miller [7] remarks that the contribution of financial markets to economic growth should not be contemplated in any serious discussions at sophisticated fora. However, other economists in the late 1800s and the 1900s argued that excluding the role of the financial system in any discourse on economic growth will give a limited and myopic view of the phenomenon [8–11].

The financial sector ensures efficient allocation of financial resources by facilitating the transfer of financial resources to undertake productive investments in an economy. Hence, a well-functioning financial sector is needed to facilitate investment in the real sector, which ultimately leads to growth in economies [9]. In the financial intermediation process, financial intermediaries also reduce information asymmetry and carry the burden that individual investors will encounter in assessing firms, managers, and conditions in the macroeconomic environment [12]. Due to this function, they can identify and direct funds to entrepreneurs with higher chances of introducing a new production process and goods [13, 14]. Furthermore, financial markets also play a key role in corporate governance [15]. The monitoring role of banks through debt covenants with firms strengthens corporate governance structures. Also, the monitoring role of financial intermediaries reduces the cost of the monitoring to the individual investors as it becomes the delegated monitor, saving the costs that the individual investors would have to pay, thereby reducing aggregate monitoring costs and eliminating the free-rider problem because the intermediary is doing the monitoring on the investors’ behalf [12]. Aside from economising the monitoring process, Bencivenga and Smith [16] show that institutional monitoring by financial intermediaries ensures that capital rationing is reduced, resulting in increased productivity and growth. Furthermore, financial intermediaries play a key role in ameliorating risk, facilitating exchanges, and pooling savings which, in turn, results in growth in the real sector.

In sharp contrast, Robinson [17] pioneered in his model of expanding economy that the growth in the financial sector will depend on the performance of the economy. In support of this, Gurley and Shaw [18] argued that in periods of economic growth, countries also experience rapid growth in financial assets. Further, they averred that the development of the financial sector is contingent on conditions of supply and demand of financial assets which are very sensitive to economic development. Likewise some authors also proffer that the financial sector may be an important indicator of economic growth and not necessarily the cause of growth. For instance, Rajan and Zingales [19] divulged that financial markets can anticipate future economic growth and respond in like manner. Thus, the theoretical channels on finance-growth literature have become complex and puzzling. In analysing these contrasting perspectives, Patrick [20] coined the term “demand-following” to explain the phenomenon in which the financial sector develops in response to growth from the real economy and “supply-leading” to represent the condition where a sustained real economic growth is driven by finance. Further, he proposed the “stage of development hypothesis” to explain the interrelationship in the supply-leading and demand-following phenomena. According to Patrick [20], by supporting innovative investments, the financial sector becomes a key driver to real economic growth as countries kick-start modern growth. However, as real growth in the economy persists, the impetus of the financial sector gradually diminishes and the demand-following dominates the relationship.

In the quest to empirically examine and ascertain the validity of these channels, several conflicting results have been documented in the contemporary literature. However, since empirical results on the phenomenon can be sensitive to proxies for the financial sector [21], an underlying issue that may explain disparities in the results is the absence of a consensus on whether market-based or bank-based measures provide an accurate proxy for the financial sector. On the one hand, financial markets permit greater diversification because they provide a richer set of tools to manage risks. As economies are maturing, the need for richer risk management tools and several investment vehicles become blatant, hence a legal and regulatory environment that encourages the development of a market-based system is prioritised [12], and therefore, a market-based measure correctly captures the financial sector of emerging economies. On the other hand, the liquidity that stock markets provide can adversely affect the allocation of resources. Shleifer and Summers [22] show that liquid markets create incentives for unhealthy takeover attempts that are socially inexpedient. Moreover, excess liquidity in capital markets may encourage an investment climate where investors, in an attempt to avoid the costly and careful process of monitoring, sell their shares inexpensively. This hinders corporate governance and induces inefficiencies in resource allocation. Consequent to this, a bank-based system offers better insights into the contributions of the financial sector to economic growth [23, 24]. As a result, a potpourri of measures such as broad money, domestic credit to the private sector, venture capital availability, ease of access to loans, stock market turnover, market capitalization, and other stock market indices and bank-based indices have been used as proxies for the financial sector development to discriminate the finance-growth nexus [21, 23–28].

Despite the arguments purporting to show the superiority of one measure over the other, stock markets and banks may play a complementary role in enhancing economic growth [29–32]. Corollary to this, a nascent and fledgling body of literature has directed their attention towards using the financial sector indices of countries [33–35] to measure the overall performance of the financial sectors of economies. Although the indices are a market-based measure, it captures the performance of banks, insurance services, real estate investment trusts, capital markets, and other providers of financial services whose services are germane to the financial sector and matter for risk diversification, resource allocation and dealing with information asymmetry. This is important because the different contributions of the various actors could have synergistic benefits on economic growth [36]. Hence, the financial sector indices enable us to capture the multidimensionality of the financial sectors of the economies of BRICS. Thus, we proxy the performance and growth of the indices which capture a broad array of firms offering financial services as a measure of financial development.

This paper offers different insights into the finance-growth nexus in two ways. First, we employ the financial sector index as a proxy for the financial sectors of the BRICS economies. This enables us to disentangle the contribution of the financial sector from the real sector in the finance-growth nexus and still capture the performance of banks and other providers of financial services. Second, we explore the time- and frequency-varying relationship between the financial sector index and economic growth in emerging markets with a bi-wavelet, partial wavelet, and wavelet multiple correlations (WMC). The extant literature that examines the relationship between the financial sector and economic growth assesses this phenomenon with less concentration on time-frequency analysis which has the potential to influence the results [32, 37–41]. However, the intrinsic complexity in time series analysis has increased the time-frequency domain, thus, wavelet analysis is becoming a usual instrument for examining confined variations of power within a time series to define both prevailing modes of variability and how the modes change in time through decomposition. Thus, the application of bivariate, partial and wavelet multiple correlations (WMC) is important in this study. While bivariate wavelet coherence is a technique that shows the correlation between two variables, partial wavelet shows the comovements between two variables relative to a common interdependence, and the resulting wavelet transformation coherence of several variables is ideal for WMC [42]. Specifically, we seek to examine the lead-lag relationships at the diverse time and frequency domains using the bivariate and partial wavelet analysis. This will enable us to assess the time and frequency at which two variables lead or lag and further control for a common interdependence. We additionally assess the degree of integration between all the variables simultaneously for each of the selected emerging economies by applying the WMC. Thus, the WMC will be used to determine the leading/lagging variable relative to the scales through linear combinations for more than two variables to provide the full picture of the nexus [43].

In particular, we examine the time-varying relationship between the financial sector and economic growth and control for external uncertainty shocks for several reasons. The stage of development hypothesis implies that the finance-growth nexus could be time-varying. Specifically, Patrick [20] divulged that, for a start, the financial sector would drive economic growth, but such impetus will diminish over time and then, economic growth will lead the relationship. Similarly, Grochowska, Diaconescu, Margerit, and Tomova [44] proffered that the relationship between finance and growth is complex, unstable over time, and varies over the cycle. Furthermore, external uncertainty may undermine activities of the financial sector and can be detrimental to growth in BRICS economies. Bhattarai, Chatterjee, and Park [45] note that external uncertainty shocks exhibit significant uncertainty spillovers to emerging economies. Previously, Horvath and Zhong [46] had documented that external shocks have a sizeable impact on macroeconomic fluctuations in emerging countries and that a considerable fraction of this impact is through the domestic stock market.

Global policy uncertainty can negatively affect banks’ liquidity creation [47] and negatively influence stock market performance [48] which can hurt the stability of the financial sector [49] in BRICS. In another vein, economic policy uncertain can have severe implications on investments, consumption and employment [47] and consequently economic growth [50]. Thus, in analysing the time-varying dynamics in the finance-growth nexus, we also note that global uncertainty shocks can affect the comovements between the financial sector and economic activity, and has implications on the extent to which the financial sector indices and economic growth leads/lags in a linear combination at multiscales (short-, medium-, and long-term). Accordingly, the partial and wavelet multiple techniques accurately delineate the interdependencies demonstrated by the finance-growth dynamics in the midst of global economic policy uncertainty shocks.

We focus on examining the time-varying relationships between the financial sector index and economic growth in BRICS (Brazil, Russia, India, China, and South Africa) for two key reasons. First, these countries are considered among the leading emerging economies in the world and are also viewed as building blocks for innovation. Although these economies have experienced recent development in their financial sectors, their financial sectors still lag behind that of other developed countries. Second, the economies of the BRICS countries have received little attention on the finance-growth nexus. The long-aged debate on the finance-growth nexus in the BRICS countries has largely been some other stock market-based or bank-based discussion, paying limited attention to the financial sector index, and the time-varying dynamics in the relationships. In our attempt to fill this gap, we note that these five countries command about 42% and a third of the world’s population and GDP respectively. Furthermore, they represent the leading emerging countries across several continents (Africa, Asia, Europe, and South America), and hence, the economies of BRICS provide valuable insights into the global emerging market economy. Accordingly, this paper investigates the time and frequency lead-lag relationships between the financial sector index and economic growth. We also control for a common interdependence (global economic uncertainty shocks) and examine the degree of integration among all the variables for each of the BRICS economies.

Our empirical results show that the financial sector indices and economic growth exhibit a bi-directional causality. Furthermore, we document overwhelming evidence that suggests that economic policy uncertainty significantly influence the relationship between the financial sector indices and economic growth as indicated by the partial wavelet. Moreover, the outcome from the wavelet multiple cross correlations indicates that the RFSI is the first mover at most time scales for the BRICS economies. We confirmed from the findings that the COVID-19 pandemic had an adverse impact on the contribution of RFSI to GDP when the pre-COVID-19 pandemic analysis was conducted for the multiple wavelet. Consequently, it was clear that the GEPU had less tendency to drive the RFSI and GDP for all economies in the pre-COVID-19 pandemic sample. In this sense, our study demonstrates that the relationship between the financial sector index and economic growth is time-varying and must be observed with external uncertainty shocks in perspective.

The next of this paper reviews brief literature on existing empirical studies. This is followed by a discussion of the methodology employed in the study. Further, the study also presents and discusses the findings and finally, provides some implications for policy and practice.

## 2. Literature review

The quest to ascertain a relationship between the financial sector and economic growth began as early as the 18th century. However, the modern empirical literature on the finance-growth nexus developed from the 1900s. The theoretical linkages have already been discussed and therefore for want of space, we present a summary of the extant empirical studies that have examined the phenomenon. These studies, which have mainly tested the direction of the causality between the variables have resulted in competing results and hypotheses.

The first strand of studies has given empirical support to the supply-leading hypothesis, where the financial sector is the main driver of economic growth. Notable amongst such are the studies of King and Levine [13], Levine, Loayza, and Beck [51], Chortareas et al. [52]. The demand-following hypothesis, which also argues that real economic growth is the driver of the financial sector has also received several empirical backing [53–55]. Furthermore, Chortareas et al. [52] documented evidence of a bi-directional relationship between the financial sector and economic growth in emerging markets. This is broadly classified as the feedback hypothesis [52, 55–58]. In addition, the neutrality hypothesis, which states that there is no relationship between the financial sector and the banking sector has also been given attention in the extant literature [59]. In the wake of the disparities in the extant literature on the phenomenon, Vogiazas, and Nellis [60] argue that the exact role played by the financial sector in the finance-growth nexus is uncertain and complex. Although most of the aforementioned studies investigated the finance-growth nexus with panel estimation techniques, the relationship between the financial sector and economic growth may depend on country-specific specific characteristics [61].

Empirical evidence on the finance-growth nexus in BRICS countries reveals contrasting findings. In Brazil, Stefani [38] employed a vector autoregressive (VAR) model and documented evidence of finance-led growth. Da Silva [62] also employed a spatial-autoregressive disturbance model and documented that the financial sector in Brazil is positively linked to growth in the area. Using a non-linear ARDL model, Nyasha and Odhiambo [63] found evidence to conclude that that the stock markets in Brazil drive growth rather than the banking sector. This was also confirmed by Moyo et al. [37] who found stock market variables to affect economic growth in Brazil. For Russia, Ono [39] used a VAR model and documented evidence of causality from economic growth to money supply and bank lending (demand-following) hypothesis. Conversely, Ono [64] had found evidence of a supply-leading hypothesis. Similarly, Fidrmuc, Fungáčová, and Weill [65] revealed that bank’s liquidity creation positively affected growth in Russia. In India, several studies have recorded evidence to support a finance-led growth [66, 67] and the supply-leading hypothesis [68, 69]. However, Nain and Kamaiah [70] do not find any evidence of causation. Concerning China, the empirical evidence from the extant literature is also not so different from those garnered from Brazil, Russia and India. Pan and Mishr [23] found evidence of a growth-led stock market development in China. However, a meta-analysis performed by Guo and He [71] divulge that the banking sector is a key driver of economic growth than the stock market in China. Meanwhile, Tang [72] documents no evidence of a finance-led growth in China but contingent on industrialization. Similarly, Sunde [73] revealed evidence of bi-directional causality between financial development and economic growth in South Africa. Meanwhile, Odhiambo [74] had earlier rejected any possibility of a supply-leading relationship and concluded overwhelming evidence for a demand-following hypothesis. Further, Nyasha and Odhiambo [75] showed that bank-based measures exhibited a positive relationship with economic growth while market-based financial development had no relationship with growth in South Africa.

Although it is widely accepted in the literature that the financial sector is important to the economy. The summary of some empirical studies conducted in BRICS confirms that it is still unclear the exact contribution of the financial sector to economic growth. The disparities in existing empirical evidence could be because the relationship between the financial sector and economic growth is not stable over time [44, 56]. Moreover, Guru and Yadav [21] show that proxies for the financial sector also explains differences in existing results. The extant literature has discriminated against the finance-growth nexus with various bank-based and other stock market-based proxies with little attention paid to the financial sector indices. In this regard, we attempt to explain how the relationship between the financial sector and economic growth varies over the business cycles and financial cycles in BRICS countries. We examine the lead-lag relationships at a different time and frequency domains using the bivariate wavelet analysis. We also note that the extent of global uncertainty shocks could affect economic activity and the financial sector [76] and has implications on the extent to which the financial sector indices and economic growth comove. In consequence of that, we also employ the partial wavelet analysis to assess the time and frequency at which two variables lead or lag and further control for a common interdependence. Finally, we assess the degree of integration between all the variables simultaneously for each of the selected emerging economies by applying the wavelet multiple correlations.

## 3. Methodology

The study examines the time-frequency co-movement between financial sector index and economic growth in BRICS using both Bivariate, Partial and Wavelet Multiple Analysis. In the following pages, we address the continuous wavelet transform (CWTs), Partial wavelet and “maximal overlap discrete wavelet transform” (MODWT) which is the initial point of the wavelet multiple correlations (WMC) and cross-correlations (WMCC) as indicated by Gençay et al. [77].

### 3.1. Bivariate wavelet analysis

#### 3.1.1. Continuous wavelet transform (CWT)

The fundamentals of wavelet analysis comprise two factors: time or location (*i*) and scale (*s*) expressed below:

$$\psi_{ί,s}(t)=\sqrt{\frac{1}{s}}\psi (t-i)(\frac{1}{s}),\psi (•)\in L^{2}\;(R)$$ (1)

where $\sqrt{\frac{1}{s}}$ is the normalization factor, guaranteeing that the unit variance of the wavelet ||ψ_*i*,*s*_(*t*)||^2^ = 1; *i* || is the location factor, offering the precise place of the wavelet; and (*s*) is the scale dilation factor, describing the stretched nature of the wavelet. The Morlet wavelet can be precise below

$$\varphi^{M}(t)=\pi^{-1/4}e^{i\omega_{o}t}e^{{-t}^{2}/2}$$ (2)

where the dominant frequency of the wavelet is denoted by *ω*_*o*_. We set *ω*_*o*_ at 6 Rua and Nunes [78].

A time series *x (t)* based on a selected mother wavelet can be decomposed as:

$$w_{x}(i,s)=\int_{-\infty }^{\infty }x(t)\sqrt{\frac{1}{s}}\psi (\frac{t-i}{s})dt$$ (3)

By sticking out the specific wavelet ψ (•) onto the designated time series, we certainly attain *w*_*s*_(*i*,*s*). Compatibly, the key benefit of a CWT is its facility to decompose and recreate the function *x*(*t*) ∈ *L*^2^(R)

$$x(t)=\frac{1}{C_{\varphi }}\int_{0}^{\infty }[\int_{0}^{\infty }W_{x}(i,s)\psi_{ί,s}(t)di]\frac{ds}{s^{2}},s>0$$ (4)

#### 3.1.2. Wavelet transform coherence

The squared absolute value of a wavelet cross-spectrum normalization to a single spectrum of wavelet power is well known as the WTC [79]. As a result, the squared wavelet coefficient is stated as follows

$$R^{2}(x,y)=\frac{{\left|\rho (\frac{1}{s}W_{xy}(i,s))\right|}^{2}}{{\rho (\frac{1}{s}|W_{x}(i,s)|}^{2}){\rho (\frac{1}{s}|W_{y}(i,s)|}^{2})}$$ (5)

where *ρ* indicates a smoothing factor, which balances resolution and significance, and $0\leq R_{xy}^{2}(i,s)\leq 1$. A value near to 0 specifies a weak relationship, while a value near to 1 designates a strong co-movement. There is a complete co-movement between the series in the time-frequency domain depicted by wavelet analysis. A stronger dependency is demonstrated by a hotter colour. The statistical implication of the relationship is scrutinised by Monte Carlo procedure since the theoretical distribution of the cross wavelet transform coefficient is unknown [79].

#### 3.1.3. WTC phase difference

The wavelet transforms coherence Phase difference indicates the interruptions in the oscillation concerning the observed time series. Following Bloomfield et al. [80], the phase difference between *x*(*t*) and *y*(*t*) is characterised as below

$$\emptyset_{xy}(i,s)=\tan^{-1}\left(\frac{J\{S(\frac{1}{s}W_{xy}(i,s))\}}{R\{S(\frac{1}{s}W_{xy}(i,s))\}}\right)$$ (6)

where J and R are the imaginary operators and real operators respectively. In the wavelet coherence map, the dimensional phase pattern outlines the influence of the wavelet coherence difference. The dimensional arrows are utilised to differentiate diverse phase patterns.

### 3.2. Partial wavelet coherence (*PWC*)

Partial Wavelet Coherence can help solve the problem of “pure” correlation between international stock markets and eliminate the influence of time series *z(t)* on the wavelet coherence between *x*(*t*) and *y*(*t*) [81].

Partial Wavelet Coherence can be defined using an equation similar to the partial correlation squared, as follows

$$R_{p}^{2}(x,y,z)=\frac{{\left|R(x,y)-R(x,z)⦁{R(x,y)}^{*}\right|}^{2}}{{\left[1-R(x,z){]}^{2}[1-R(y,z)\right]}^{2}}$$ (7)

where $R_{p}^{2}(x,y,z)$ ranges from 0 to 1. In this paper, *x* and *y* denotes the financial sector index returns and economic growth returns while *z* denotes GEPU. A Monte Carlo methods are used to estimate PWC.

### 3.3. Wavelet multiple correlation (WMC)

Let *X*_*t*_ = *x*_1*t*_, *x*_2*t*_,…,*x*_*nt*_ be a multivariate stochastic process and let *W*_*jt*_ = *w*_1*jt*_, *w*_2*jt*_,….,*w*_*njt*_ represent the resultant scale *λ*_*j*_ wavelet coefficients attained by employing the MODWT. Fernández-Macho [82] outlines the WMC represent by Ω*X*(*λ*_*j*_) as a set of multiscale coherence calculated from *Xt* as follows. The square roots of the regression coefficient of determination (R^2^) formed by the linear combination of *w*_*ijt*_, *i* = 1,2,…,*n* variables for which such R^2^ is maximum is calculated at each wavelet scale *λ*_*j*_. It is known from earlier studies that none of the auxiliary regressions ought to be run, since the R^2^ conforming to the regression of a variable *z*_*i*_ on a set of predictors {*z*_*k*_, *k* ≠ *i*} can be represented as $R_{i}^{2}=1–\rho^{-ii}$, where *ρ*^*ii*^ is the ith diagonal element of the inverse of the complete correlation matrix *P*. Hence WMC is achieved as in Eq (8), as

$$\Omega X(\lambda_{j})={\left(1-\frac{1}{max\;diagP_{j}^{-1}}\right)}^{1/2}$$ (8)

where *Pj* is the (n x n) correlation matrix of *W*_*jt*_

With regards to the theory of regression, and the fitted values of *z*_*i*_ as ${\hat{z}}_{t}$, then the WMC can be expressed as Eq (9)

$$\Omega X(\lambda_{j})=\frac{Corr(w_{ijt},{\hat{w}}_{ijt})Cov(w_{ijt},{\hat{w}}_{ijt})}{{\left(Var(w_{ijt})Var({\hat{w}}_{ijt})\right)}^{1/2}}$$ (9)

where *w*_*ij*_ is selected to maximize Ω*X*(*λ*_*j*_) *and* ${\hat{w}}_{ijt}$ are the fitted values in the regression of *w*_*ij*_ on the remaining wavelet coefficients at scale *λ*_*j*_.

We may define WMCC as generated by allowing a lag *τ* between observed and fitted values at each scale *λ*_*j*_ below

$$\Omega X,\tau (\lambda_{j})=Corr(w_{ijt},{\hat{w}}_{ijt+\tau })=\frac{Cov(w_{ijt},\ddot{{\hat{w}}_{ijt+\tau }})}{Var(w_{ijt})Var({\hat{w}}_{ijt+\tau })}$$ (10)

where for n = 2, WMC and WMCC converge with the standard wavelet correlation and cross-correlation.

To estimate WMC and WMCC let the realization of the multivariate stochastic process *X*_*t*_ for *t* = 1,2,…,*T* be *X* = {*X*_1_, *X*_2_,…,*X*_*T*_}. Relating a MODWT of order *J* to each of the univariate time series {*X*_1*i*_,…,*X*_1*T*_}, for *i* = 1,2,…,*n*, the *J length*−*T* vectors of coefficients of MODWT ${\tilde{W}}_{j}=\{{\tilde{W}}_{j1},{\tilde{W}}_{j1},\ldots ,W{\tilde{W}}_{j,\;T-1}\},$ *for j* = 0, 1,…,*J* is obtained.

From Eq (9), a nonlinear function of all *n*(*n*−1)/2 wavelet correlations of scale *λ*_*j*_ and a steady estimator of wavelet correlation from the MODWT can be represented by

$$\tilde{\Omega }X(\lambda_{j})={\left(1-\frac{1}{maxdiag{\tilde{P}}_{j}^{-1}}\right)}^{1/2}=\frac{Corr({\tilde{w}}_{ijt},{\hat{\tilde{w}}}_{ijt})Cov({\tilde{w}}_{ijt},{\hat{\tilde{w}}}_{ijt})}{{\left(Var({\tilde{w}}_{ijt})Var({\hat{\tilde{w}}}_{ijt})\right)}^{1/2}}$$ (11)

where ${\tilde{w}}_{ij}$: the regression of the same set of regressors $\left\{{\tilde{w}}_{kj},\;k\neq i\right\}$ maximizes the R^2^, ${\hat{\tilde{w}}}_{ij}$ denotes conforming fitted values, and *L*_*j*_ = (2^*j*^−1)(*L*−1) is the number of wavelet coefficients influenced by the boundary conditions associated with wavelet filter of length *L* and scale *λ*_*j*_ but $\tilde{T}=T-L_{j}+1$ is the number of wavelet coefficients unaffected by the boundary conditions.

In the same vein, a consistent estimator of the WMCC can be computed as

$$\tilde{\Omega }X,\tau (\lambda_{j})=\frac{Corr({\tilde{w}}_{ijt},{\hat{\tilde{w}}}_{ijt+\tau })Cov({\tilde{w}}_{ijt},{\hat{\tilde{w}}}_{ijt+\tau })}{{\left(Var({\tilde{w}}_{ijt})Var({\hat{\tilde{w}}}_{ijt+\tau })\right)}^{1/2}}$$ (12)

In calculating confidence interval (CI) of WMC, Fernández-Macho [82] applies the transformation defined as arctan *h*(*r*), where arctan *h*(.) is the inverse hyperbolic tangent function for simplicity sake Fernández-Macho [82]. The confidence interval is built on the same assumption of the realization of *X* in the estimation of WMC and WMCC and hence for $\tilde{\Omega }X(\lambda_{j})$ in Eq (11), the ${\tilde{z}}_{j}∼Fℵ(z_{j},{\left(T/2^{j}-3\right)}^{-1})$, where $z_{j}=arctan\;h(\Omega X(\lambda_{j})),\;{\tilde{z}}_{j}=arctan\;h(\tilde{\Omega }X(\lambda_{j}))$, and Fℵ symbolize the folded normal distribution. Therefore, an approximate (1 − α) CI is represented by

$$CI(1-\alpha )(\Omega X(\lambda_{j}))=tanh\;\left[{\tilde{z}}_{j}-\frac{C_{2}}{{\left(\frac{T}{2^{j}}-3\right)}^{\frac{1}{2}}};\;{\tilde{z}}_{j}+\frac{C_{1}}{{\left(\frac{T}{2^{j}}-3\right)}^{\frac{1}{2}}}\right]$$ (13)

where the Fℵ critical values *C*_1_, *C*_2_ are: Ω(*C*_1_)+Ω(*C*_1_−2*z*^0^) = 1−*α*/2 and Ω(*C*_2_)+Ω(*C*_1_−2*z*^0^) = 2−*α*/2 with Ω(.) as the standard Gaussian probability distribution function and $tanh(z^{0})=\Omega_{X}^{0}(\lambda )$ as the value of some WMC formulated under a null hypothesis of the absence of correlation.

## 4. Empirical analysis

### 4.1. Data description

The study employed monthly data on financial sector index (FSI) of BRICS economies which are made up of Brazil (Brazil IFinanceiro Index), Russia (Moscow Exchange Financial Index), India (NIFTY Financial Services Index), China (Shanghai Financials Sector Index) and South Africa (FTSE/JSE Financial 15 Index). We further consider the gross domestic product (GDP) of each country and the global economic policy uncertainty.

Owing to the quarterly release of GDP, the Organization for Economic Corporation and Development (OECD) constructed the monthly GDP to gauge its short-term dynamics. The monthly GDP is developed from chained volume estimates of quarterly GDP series in US dollars. Through a linear interpolation, the monthly GDP is obtained to align with the required month of a quarter. The data on financial sector indices were obtained from EquityRT database, economic growth data was gleaned from OECD database, and data on global economic policy uncertainty developed by Baker, Bloom, and Davis [83] was extracted from the website https://www.policyuncertainty.com/index.html. Both the GEPU and GDP are normalised to ensure stability and effective comparison over time. In addition, the GDP as provided by the OECD for each country is seasonally adjusted to deal with periodic swings in the GDP. Consequently, fluctuations that normally occur across time which includes inflation are expunged to better reflect the correct configurations in economic activity. In order to ensure a smooth comparison, we remove inflation (CPI) from the FSI observations for each country to obtain the real financial sector index (RFSI). As shown below, the study was based on monthly returns–*r*_*t*_ = ln*P*_*t*_−*lnP*_*t*−1_, where *r*_*t*_ is the continuously compounded return, *P*_*t*_ and *P*_*t*−1_ are current and previous indexes respectively. The suggested period as shown in Table 1 was chosen due to consistent data availability, hitherto it considers the COVID-19 pandemic period. The differences in sample window as shown in Table 1 do not distort the analysis of this study because each country is separately assessed to reveal its distinct finance-growth nexus dynamics. Notwithstanding, the bi-wavelet and partial wavelet techniques reveal chunks of time-frequencies which facilitate comparisons where dates overlap.

**Table 1:** Summary of sample data.

Countries	Period	Observations
Brazil	01/01/2005–01/05/2021	197
Russia	01/12/2004–01/02/2021	195
India	01/02/2012–01/05/2021	112
China	02/02/2009–01/05/2021	148
South Africa	01/02/2002–01/02/2021	254

### 4.2. Descriptive statistics

**Fig 1:**
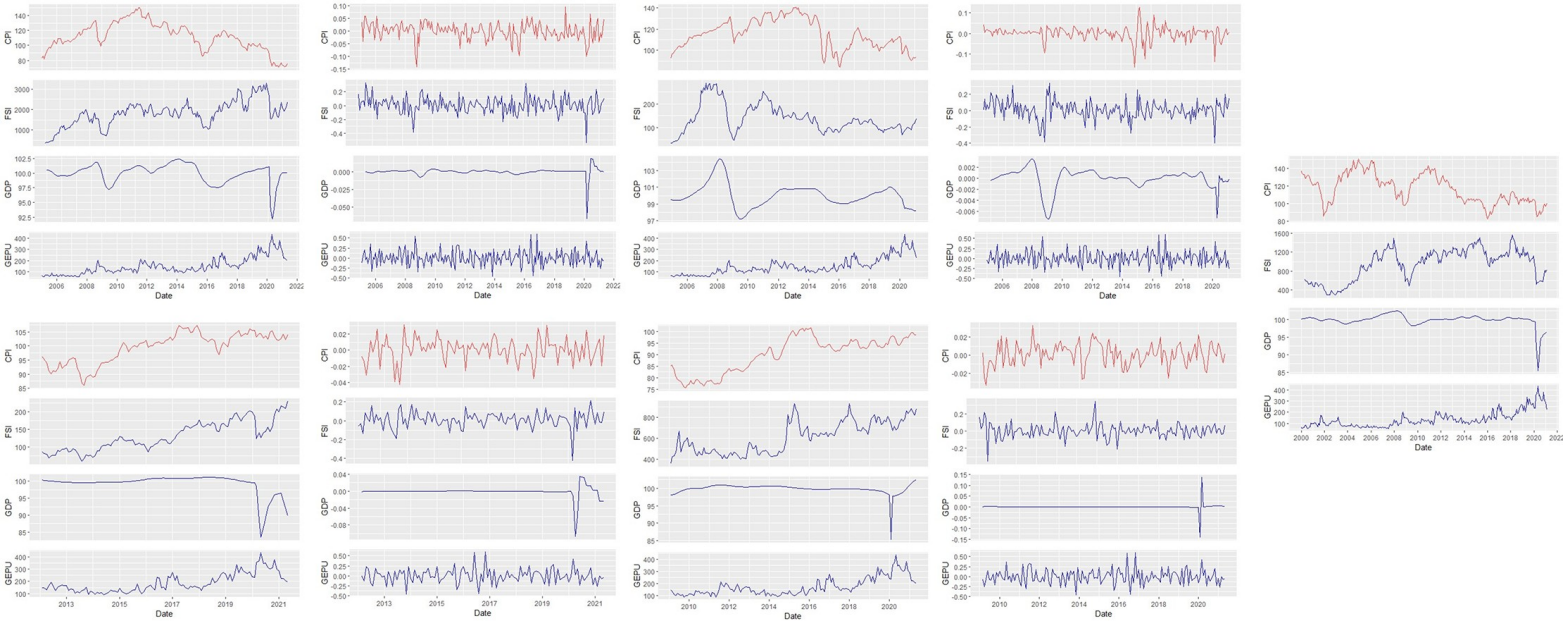
Plots of indices and returns series.

Fig 1 illustrates both the price and log-return plots of the BRICS economies for inflation (CPI), financial sector index (FSI), economic activities (GDP) and global economic policy uncertainty (GEPU). We find that the CPI of Brazil, Russia and South Africa trends downwards, and upwards for China and India. The fluctuations in inflation for each country is quite substantial, and its absence from the analysis may confound the results. The significant variations in the monthly prices are apparent in Fig 1 especially during the global financial crisis of 2007–2009, and the COVID-19 pandemic period across markets. In addition, the log-return plots exhibit volatility clustering as anticipated due to the stylised facts of financial time series.

Table 2 exhibits the summary of statistics for the CPI, financial sector index, economic growth (GDP) and global economic policy uncertainty (GEPU) during the period under study. The skewness values observed shows non-normality across board with the financial sector index of China exhibiting a skewness close to normality of -0.0268. On the other hand, kurtosis values further show leptokurtic behaviour in the values across variables especially global economic policy uncertainty. In terms of the stationarity test, the Augmented Dicky-Fuller (ADF) and the Kwiatkowski-Phillips-Schmidt-Shin (KPSS) are used. The observations from both the ADF and the KPSS reveal that all the data series explicitly fulfil the stationarity requirements. The White Neural Network test of linearity indicates that the GDP for BRICS is non-linear. This puts the time-frequency and frequency dependent analysis of this study into perspective.

**Table 2:** Summary statistics.

Statistic	CPI		Financial Sector Index	GDP	GEPU
		**Brazil**			
Mean	-0.0005		0.0095	0.0000	0.0058
Std. dev.	0.0332		0.1156	0.0058	0.1801
Skewness	-0.6877		-0.4829	-7.3576	0.3738
Kurtosis	4.7455		5.4898	89.8349	3.6167
Normtest W	0.9700***		0.9710***	0.4060***	0.9876*
***Unit Root tests***					
ADF	-4.9948***		-5.4317***	-6.0078***	-6.5585***
KPSS	0.4431*		0.1840	0.0244	0.0354
**Linearity test**					
White Neural Network test	0.4256		1.5984	13.941***	2.9379
		**Russia**			
Mean	0.0001		0.0069	-0.0001	0.0063
Std. dev.	0.0338		0.1111	0.0019	0.1809
Skewness	-1.1134		-0.3433	-2.0075	0.3658
Kurtosis	8.7743		4.7927	8.2046	3.5827
Normtest W	0.8702***		0.9693***	0.7930***	0.9880
***Unit Root tests***					
ADF	-6.5641***		-4.5688***	-4.8669***	-6.7010***
KPSS	0.2101		0.2026	0.1101	0.0269
**Linearity test**					
White Neural Network test	0.5647		2.2285	5.0292*	3.4170
		**India**			
Mean	0.0007		0.0089	-0.0010	0.0026
Std. dev.	0.0143		0.0851	0.0137	0.1831
Skewness	-0.5077		-1.0979	-4.6339	0.3378
Kurtosis	3.5585		7.8529	37.5746	4.0093
Normtest W	0.9767**		0.9355***	0.3762***	0.9802*
***Unit Root tests***					
ADF	-4.738***		-4.9826***	-5.0187***	-6.3547***
KPSS	0.0693		0.0516	0.1145	0.0673
**Linearity test**					
White Neural Network test	1.4381		3.6352	16.071***	1.9183
		**China**			
Mean	0.0010		0.0062	0.0003	0.0021
Std. dev.	0.0119		0.0829	0.0163	0.1793
Skewness	-0.2519		-0.0203	-0.3428	0.3838
Kurtosis	2.9096		6.4535	72.5828	3.7717
Normtest W	0.9932		0.9550***	0.1595***	0.9860
***Unit Root tests***					
ADF	-5.162***		-4.7286***	-6.2889***	-5.5779***
KPSS	0.0968		0.0349	0.0516	0.0488
**Linearity test**					
White Neural Network test	1.4478		0.1095	68.888***	2.3144
		**South Africa**			
Mean	-0.0012		0.0011	-0.0001	0.0051
Std. dev.	0.0342		0.0881	0.0067	0.1810
Skewness	-0.9011		-0.8821	-3.3250	0.5372
Kurtosis	5.8011		7.4071	44.9400	4.0475
Normtest W	0.9581***		0.9532***	0.2919***	0.9810***
***Unit Root tests***					
ADF	-6.3109***		-5.7217***	-5.2645***	-7.6597***
KPSS	0.0360		0.0912	0.0378	0.0251
**Linearity**					
White Neural Network test	4.4952		1.0293	6.9139**	3.344

Note: Normtest W* indicates nonnormal distribution at all conventional levels of significance.

[*], [**], and [***] indicate significance at 10%, 5% and 1% levels respectively.

### 4.3. Main results

#### 4.3.1. Bi-wavelet and partial wavelet

The statistical interpretations and the availability of codes for analysis were found in the biwavelet package provided by Gouhier et al. [84]. Right-pointing arrows and left-pointing arrows indicate when real financial sector index (RFSI) and gross domestic product (GDP) are respectively in-phase (movement in identical direction) and antiphase (movements in the reverse direction) to ensure the smooth interpretation of the results. Right-pointing arrows upwards and left-pointing arrows downwards mean that the first variable leads, while the left-pointing arrows upwards and the right-pointing arrows downwards mean that the second variable leads. The intensity of the interdependence between the paired series is indicated by the surface colour and the colour pallet is represented. The red (warm) colour signifies parts that have major interactions, while the blue (cold) colour shows a lower series correlation. The cone of influence (COI) within the bivariate plots indicates the region where interpretation of the wavelet becomes important. The results outside the COI may be distorted or unreliable because of the end-points influence of finite length signals.

**Fig 2:**
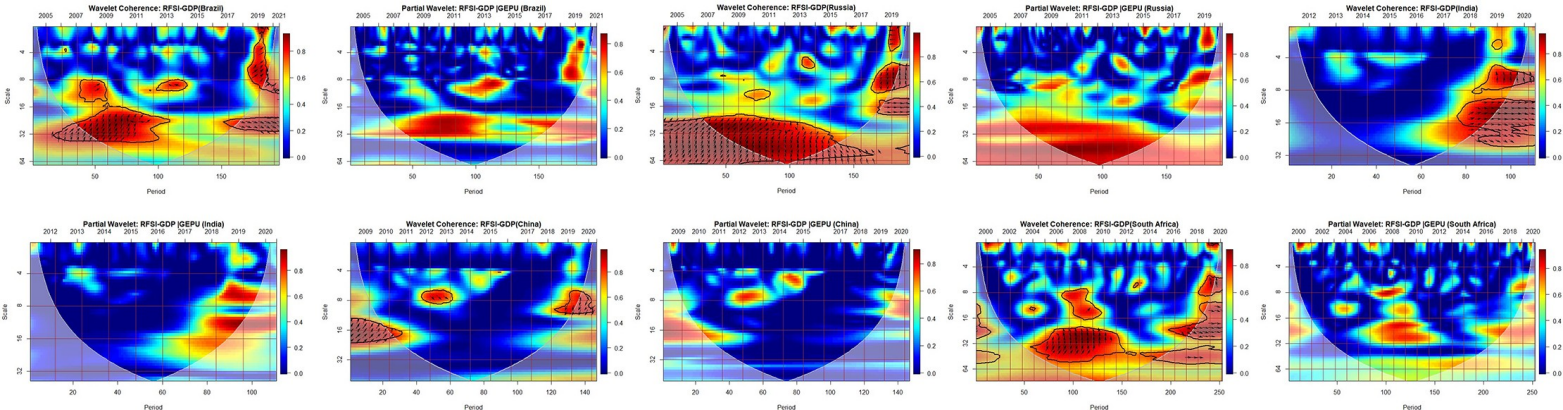
Nexus between RFSI-GDP while considering external uncertainty shocks.

Fig 2 presents the comovements between RFSI and GDP via the bivariate technique as well as the influence of GEPU on the nexus between RFSI and GDP through the partial wavelet coherence each for BRICS economies. In general, the combined influence of RFSI and GDP appears more dominating for BRICS economies except for China and possibly India from the Asian continent. The remaining BRICS economies exhibit a strong sensitivity for the period 2005–2021, mostly from the medium to the long-term domain (beyond 8 months scale). This is to say India and China demonstrate less intensity of significant influence for most time and frequency horizons. This is probably because even though China and India are in the top five biggest economies in the world, their financial sectors are still maturing [85]. Consequent to this, the size of the financial sector may not contribute immensely to commensurate the size of their GDP. More importantly, these economies rely heavily on sectors largely driven by foreign direct investment inflows [86].

The right-pointing arrows upwards as shown for Brazil, Russia and South Africa from the medium-to-long terms horizon signify that the RFSI drives economic growth during these periods. As a result, the aforementioned countries’ economic development hinges on the effective development of their financial sector. Accordingly, the development of the financial system contributes to economic growth via fund inflow by offering capital accumulation and technological development infrastructure to economies [87]. The few right-pointing arrows downwards for Russia from 2007–2010 at scales 8–16 months demonstrates that economic growth significantly drives the financial sector. The 2007–2009 Global Financial Crisis and Euro Crisis of 2010 could be a plausible attribution. Notwithstanding, the right-pointing arrows for most BRICS economies depict that the RFSI and economic growth exhibit a positive relationship between them. This suggests that as either the financial sector or economic growth is improved, it contributes to the development of the other. This is to say, there exists a positive interdependency between the financial sector and economic growth, which is mainly realised from the medium to long-term perspective for most economies. In this regard, the development of the financial system would urge global investors to maximise their returns via more productive investments [9, 10, 88]. However, China exhibits the least dependency on finance growth nexus followed by India. Nonetheless, countries with the less developed financial system have a higher likelihood of experiencing market failure issues as a result of incomplete competition and asymmetric information [89].

Following the impact of the COVID-19 pandemic on most financial markets [90], we recognise some patches of right-pointing arrows downwards from 2019 to 2021 for most economies, especially in the long-term perspective. At this point that financial markets are adversely affected as indicated by extant literature [91, 92] coupled with contagion effects from external shocks, there is a turn of event where economic growth now drives the financial sector which continues to establish the positive interdependency across time. This establishes that where economic growth increases, the demand for financial services (financial growth) is elicited by economic growth [93]. In the COVID-19 period, financial activities have been depressed with investors unwilling to pursue new investments due to uncertainty in economic conditions [94]. Consequent to this, investors and subsequent demand for financial assets and investment vehicles will be driven by positive news of economic growth.

Moreover, taking cognisance of shocks from external policy uncertainties on the relationship between RFSI and GDP, the significance of the already established relationship diminishes. That is, with the aid of the partial wavelet coherence, global economic policy uncertainty distorts the significance and directional comovements between RFSI and GDP. This signifies that the relationship between RFSI and GDP is more vulnerable to global economic policy uncertainty which the latter has been revealed by extant literature to have a significant influence on financial markets and economic activity [46, 74, 95, 96]. The details of the statistical interpretations are saved for readers, however, a more extensive review could be made with the aid of the guidance provided in the initial paragraph of this section.

In summary, we document a bi-directional causality between the RFSI and economic growth. This supports both the supply-side perspective engineered by Schumpeter [9], which posits causality from finance to economic growth and the demand-side approach led by Robinson [17] which proposes that economic growth causes financial development. The outcome of the study also supports prior empirical studies within the field of financial development and economic growth [97, 98], while other studies also indicate a unidirectional relationship [87, 99]. We, therefore, advocate that finance-growth nexus as well as causality dynamics are time-varying, and are likely to be influenced by external policy uncertainty shocks.

#### 4.3.2. Frequency-dependent analysis

We present the wavelet multiple analysis separately for each BRICS economy based on the three main indicators, which are; real financial sector index (RFSI), gross domestic product (GDP) and global economic policy uncertainty (GEPU). We use a monthly data and set, *lj*, *j* = 1…4, of the wavelet factors, which are connected to times of, respectively, “2–4 months (short term), 4–8 months (medium-term), 8–16 months (medium-term), above 16 months (long-term)” from Figs 4 and 5, following Li, Li, Yuan, and Yu [100].

*4*.*3*.*2*.*1*. *Wavelet bivariate correlations matrix*. At 5 wavelet scales, the bivariate contemporary correlations are considered. Wavelet bivariate correlations matrix. The codes for the variables are GEPU (C1), GDP (C2) and RFSI (C3). For calculating wavelet correlation coefficients, the horizontal axis displays the possible combinations. If we switch from left to right, the similarities between the pairs of FSI, GDP and GEPU nexus become weaker. On the vertical axis, the wavelet scales reflect periods. The purpose of the bivariate contemporary correlation matrix in this paper addresses the correlation between the realizations of two possible combinations of time series in the same period (wavelet scale). Thus, the bivariate contemporary correlation matrix is frequency (scale) dependent other than having the features of both time-frequency domains as illustrated in Fig 2.

**Fig 3:**
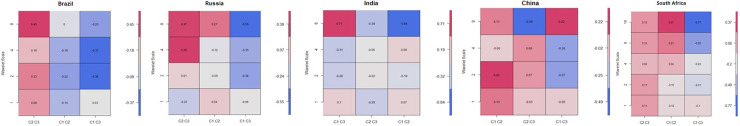
Wavelet bivariate correlations matrix. Wavelet bivariate correlations matrix. The codes for the variables are GEPU (C1), GDP (C2) and RFSI (C3).

From Fig 3, we present the wavelet correlation matrix for RFSI, GEPU and GDP returns across the five wavelet scales, which does not seem to differ significantly from the bivariate analysis from Fig 2. We find a mix of positive and negative relationships among the pairs. The RFSI and economic growth of Brazil demonstrate the maximum degrees of co-movement with coefficients fluctuating over 0.19 to 0.45 at diverse time scales averaging 0.31 with minimal extreme correlational values. This is followed by Russia, India, South Africa and China for the RFSI and GDP in the short-, medium-, and long-terms. However, the RFSI and GEPU of India seem to be highly correlated at 0.71 in the long-term horizon, with less correlation between the financial sector and economic growth. This pattern of dynamics of less connectedness among RFSI, GEPU and GDP from the short-medium terms (2–16 months) does not deviate from China. This means that, on average, India and China from the Asian continent seem to have low interdependencies between their RFSI and GDP, and a little high dependency with external uncertainty shocks relative to other BRICS economies. This is not startling as China and India have demonstrated similarities in many respect [101]. This developed relationship should be of much concern to investors and policymakers within this region to continuously monitor the patterns of change between these variables over time. The overall correlation analysis for each of the BRICS economies is attached as part of supporting information to the paper. Notwithstanding, evidence from the Wavelet bivariate correlations matrix contributes to the reasons why financial time series are not static.

**Fig 4:**
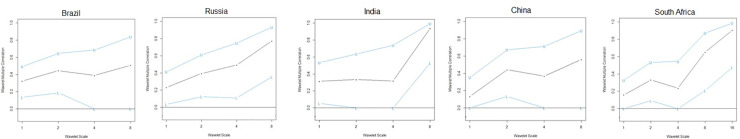
Wavelet multiple correlations among FSI, GDP and GEPU in BRlCS. U-upper limits, L- lower (at 95% confidence interval).

**Fig 5:**
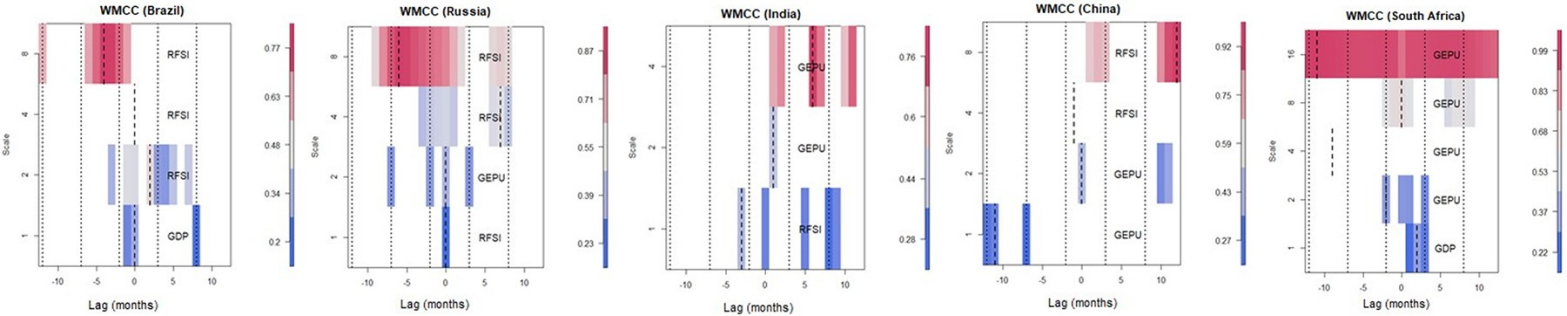
Wavelet multiple cross-correlation among RFSI, GDP and GEPU in BRICS.

*4*.*3*.*2*.*2*. *Wavelet multiple correlations (WMC)*. Fig 4 (Table 3) and Fig 5 (Table 4) denote the wavelet multiple correlations and wavelet multiple cross-correlations respectively for the FSI, GEPU and GDP nexus returns series into frequency localization by the MODWT [82].

**Table 3:** Wavelet multiple correlations (WMC).

Scale	WMC “lower”	Correlation	WMC “upper”
**Brazil**			
1	0.1348	0.3245	0.4913
2	0.1869	0.4447	0.6452
3	0.0000	0.3897	0.6854
4	0.0000	0.5077	0.8376
**Russia**			
1	0.0344	0.2323	0.4126
2	0.1241	0.3943	0.6101
3	0.1105	0.4920	0.7471
4	0.3544	0.7714	0.9325
**India**			
1	0.0523	0.3132	0.5342
2	0.0000	0.3348	0.6341
3	0.0000	0.3177	0.7393
**China**			
1	0.0000	0.1347	0.3538
2	0.1326	0.4419	0.6727
3	0.0000	0.3674	0.7121
4	0.0000	0.5595	0.8921
**South Africa**			
1	0.0000	0.1588	0.3246
2	0.0897	0.3301	0.5342
3	0.0000	0.2355	0.5444
4	0.2087	0.6513	0.8725
5	0.4758	0.9047	0.9860

**Table 4:** Wavelet multiple cross correlations (WMCC).

Scale	Localizations	Time Lag (months)	Leading/Lagging variable
**Brazil**			
1	0.3245	0	GDP
2	0.5188	2	RFSI
3	0.3897	0	RFSI
4	0.7662	-4	RFSI
**Russia**			
1	0.2323	0	RFSI
2	0.3943	0	GEPU
3	0.5346	7	RFSI
4	0.8747	-7	RFSI
**India**			
1	0.4105	-3	RFSI
2	0.4410	1	GEPU
3	0.7554	5	GEPU
**China**			
1	0.3067	-11	GEPU
2	0.4419	0	GEPU
3	0.3871	-1	RFSI
4	0.9160	12	RFSI
**South Africa**			
1	0.3471	2	GDP
2	0.3475	-2	GEPU
3	0.3127	-9	GEPU
4	0.6513	0	GEPU
5	0.9880	-11	GEPU

Fig 4 and Table 3 establish the degree of integration between the study variables (FSI, GDP & GEPU) from the short-term to the long-term dynamics in a continuous fashion. It does not necessarily indicate which variable is leading or lagging, but the overall connection of the study variables. The degree of integration is comparatively high for the monthly return series reaching as high as approximately 0.9047 (South Africa) for the wavelet multiple correlations, 0.0000 (most BRICS economies) for the lower panel and 0.9860 (South Africa) for the upper panel. There is a continuous increment in multiple correlations over the horizon for only Russia and South Africa. Generally, there is very low integration between FSI, GDP and GEPU from the short-long term, except South Africa which recorded multiple correlations as high as above 90%. Thus, from South Africa, monthly returns of one variable can be explained by the remaining two (2) variables to a degree of about 90% from monthly, leading up to scale 16 months interdependence.

*4*.*3*.*2*.*3*. *Wavelet multiple cross correlations (WMCC)*. The wavelet multiple cross-correlation coefficients are presented in Table 4 depicting four wavelet scales. From Fig 5, the scales at the y-axis have similar meanings as indicated at the preliminary stage of the wavelet multiple analysis discussion. Following Asafo-Adjei, Adam and Darkwa [102], the x-axis, however, represents the lag length of the series. In this case, 12 months for positive and negative lags each. We need both positive and negative to confirm the potential leading and lagging variables. Localisations at positive lag denote lagging variable and negative lag denote leading variable at the respective scales. At the zero-lag of localisation (dashed) lines, there is no lead or lag. Localisation implies the maximum values in the linear combination of all variables at the wavelet scales, which are indicated by dashed lines within the dotted lines (at all lags). A variable listed on a scale indicates the variable with the potential to lead or lag all the other variables. It implies that, at that scale, it has the maximum value in the linear combination of all the variables at the respective scales. When a dashed line accompanies a listed variable in the heat map, then it becomes an actual lead (negative lag) or lag (positive lag) unless the dashed line is on the zero-lag which implies neither lead nor lag. Accordingly, the economic implication of the wavelet multiple cross-correlations (WMCC) is that it indicates the degree of interdependence between the variables, and determines the most influential variable at a specified wavelet scale to act as either a leading (first mover to respond to shocks) or lagging (the last variable to respond to shocks after the remaining variables) variable.

We find that the RFSI maximizes the multiple cross-correlations from a linear combination of the remaining variables (GDP and GEPU) at diverse scales for Brazil, Russia, China and India. Specifically for Brazil, the RFSI lags (at time 2 months) in the medium-term at scale 2, but leads (at times -4months) from the long-term at scale 4. On the other hand, the RFSI in Russia has the potential to lead/lag (at time 0 month) in the short-term, lags (at time 7 months) in the medium-term, and leads (at time -7 months) in the long-term. Further, there is no leading nor lagging RFSI in South Africa for the short-, medium-, and long-terms. The RFSI leads in the short-term for India, but in the medium-term for China. This indicates that the RFSI is a first mover at most time scales for most BRICS economies. Again,

This is followed by GDP which can be found at only one scale for Brazil and South Africa. Specifically, economic growth has the potential to lead the RFSI and GEPU for Brazil (at time 0 months), but lags for South Africa (at time 2 months). This clearly indicates that GDP does not maximise the multiple cross-correlations from a linear combination relative to the remaining variables for most economies.

It can be seen from the wavelet multiple cross-correlation plots in Fig 5 and Table 4 that global economic policy uncertainty leads or lags the RFSI and economic growth for the BRICS economies except Brazil. The impact of GEPU on FSI and GDP is worst for South Africa in about four cases in the medium-, and long-terms. This signifies that South Africa’s financial markets and economic growth are vulnerable to external uncertainty shocks. However, GEPU lags for India, but has a potential to lead or lag for Russia and China in the medium-term. Cui et al. [103] observed that external uncertainty shocks has the potential to undermine corporate innovativeness in China as investments in innovation become stifled.

#### 4.3.3. Robustness check for COVID-19 pandemic shocks

We further present a confirmatory analysis to establish whether the identified degree of integration between the RFSI and GDP in the midst of GEPU are mostly driven by the COVID-19 pandemic shock. We take insights from the World Health Organization’s (WHO) communiqué, which designated the COVID-19 outbreak a Public Health Emergency of International Concern (PHEIC) on January 30, 2020, and a pandemic on March 11, 2020. We, therefore, segregate the data based on the announcement by the WHO on March 11, 2020 as a global pandemic where the Pandemic had reached its peak, and largely impacted most economies. Consequently, analysis below are conducted prior to March, 2020.

**Fig 6:**
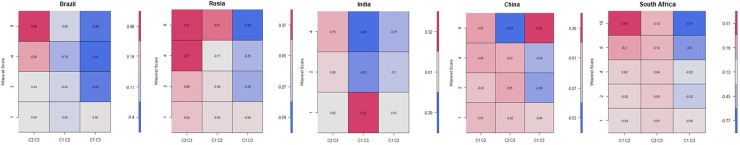
Wavelet bivariate correlations matrix. The codes for the variables are GEPU (C1), GDP (C2) and RFSI (C3).

It can be observed from Fig 6 that the relationship between the RFSI and GDP has improved as compared to the outcome of the full sample. We also find that the negative impact of GEPU on RFSI and GDP has minimised. This is to say, the COVID-19 pandemic has a significant impact on RFSI and GDP which heightens the adverse impact of GEPU as found in the full sample case.

**Fig 7:**
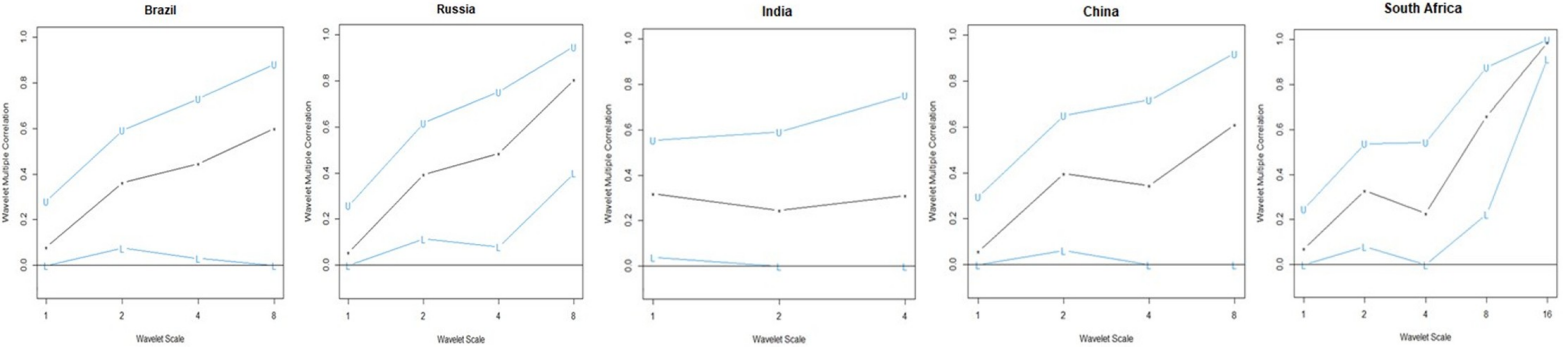
Wavelet multiple correlations among FSI, GDP and GEPU in BRlCS. U-upper limits, L- lower (at 95% confidence interval).

Fig 7 (Table 5) provides that the degree of integration between the RFSI and GDP in the midst of GEPU has improved for the BRICS economies, except India which is developing its financial sector. This indicates that prior to the COVID-19 pandemic, the contribution of RFSI to GDP was effective despite the adverse impact of GEPU. Specifically, the degree of integration for South Africa is quite phenomenal with high convergence in the long-term.

**Table 5:** Wavelet multiple correlations (WMC).

Scale	WMC “lower”	Correlation	WMC “upper”
**Brazil**			
1	0.0000	0.0793	0.2818
2	0.0760	0.3615	0.5922
3	0.0303	0.4462	0.7304
4	0.0000	0.5993	0.8821
**Russia**			
1	0.0000	0.0557	0.2587
2	0.1145	0.3948	0.6168
3	0.0789	0.4844	0.7524
4	0.3990	0.8060	0.9477
**India**			
1	0.0378	0.3185	0.5526
2	0.0000	0.2456	0.5905
3	0.0000	0.3113	0.7510
**China**			
1	0.0000	0.0580	0.2959
2	0.0607	0.3958	0.6507
3	0.0000	0.3436	0.7172
4	0.0000	0.6092	0.9192
**South Africa**			
1	0.0000	0.0705	0.2467
2	0.0807	0.3279	0.5371
3	0.0000	0.2269	0.5428
4	0.2228	0.6598	0.8760
5	0.9128	0.9872	0.9982

**Fig 8:**
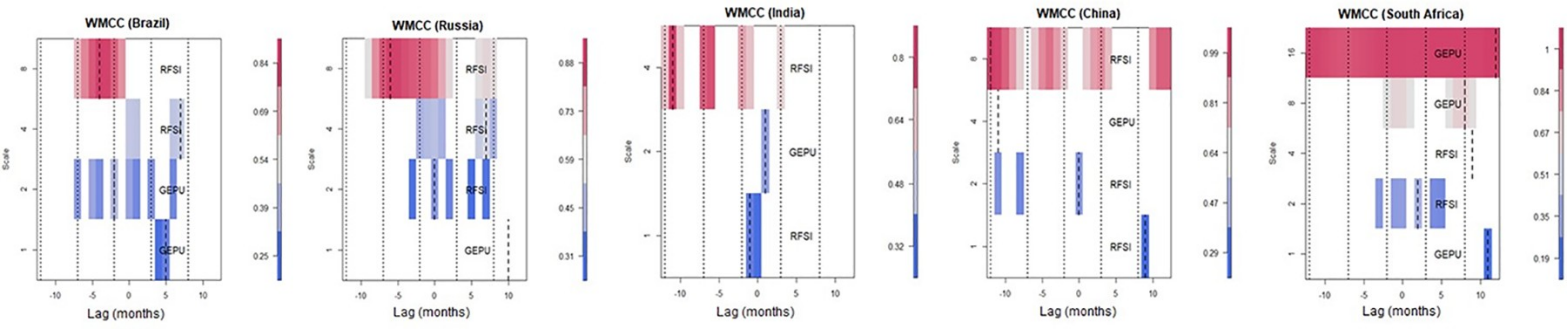
Wavelet multiple cross-correlation among FSI, GDP and GEPU in BRICS.

It is clearly shown in Fig 8 (Table 6) that the tendency for GEPU to drive the RFSI and GDP has minimised for all economies relative to the full sample scenario. Consequently, we see the RFSI acting as a first mover in the long-term for the BRICS economies, except South Africa. Thus, in line with the arguments of Aghion, Howitt, and Levine [104], we confirm that the financial sector exhibits first-order impact on economic growth by shaping the severity of credit constraints and the rate of technological innovation [105]. Also, the RFSI maximizes the multiple cross-correlations from a linear combination of the remaining variables (GDP and GEPU) at diverse scales. This indicates that the COVID-19 pandemic has distorted most economic activities and heightened the level of uncertainties rendering effective assets allocation and portfolio diversification as part of the optimum choice for investors.

**Table 6:** Wavelet multiple cross correlations (WMCC).

Scale	Localizations	Time Lag (months)	Leading/Lagging variable
**Brazil**			
1	0.2820	5	GEPU
2	0.4147	-2	GEPU
3	0.4530	6.5	RFSI
4	0.8375	-4	RFSI
**Russia**			
1	0.1938	10	GEPU
2	0.3948	0	RFSI
3	0.5407	8	RFSI
4	0.8760	-6	RFSI
**India**			
1	0.3442	-1	RFSI
2	0.4055	1	GEPU
3	0.8020	-11.5	RFSI
**China**			
1	0.2938	9	RFSI
2	0.3958	0	RFSI
3	0.4086	-11.5	GEPU
4	0.9872	-12	RFSI
**South Africa**			
1	0.1908	11	GEPU
2	0.3856	2	RFSI
3	0.2529	8.5	RFSI
4	0.6873	8	GEPU
5	0.9966	12	GEPU

## 5. Conclusion and policy implication

The analysis of the study was performed with the aid of three econometric techniques via wavelet. These are the bivariate, partial and wavelet multiple correlations analysis. We, therefore, contribute to existing knowledge on time-frequency domain co-movement between RFSI and GDP, while incorporating shocks from global economic policy uncertainty (GEPU). The techniques enabled us to determine the lead-lag relationships between the study variables and to assess their interdependencies toward investment decisions, especially, portfolio diversifications, as well as for policy decision making.

Findings from the bivariate analysis suggest a bi-directional causality between RFSI and GDP. This supports both the supply-side perspective, which hypothesises causality from finance to economic growth and the demand-side approach which proposes that economic growth causes financial development. Again, there exists a positive interdependency between the financial sector and economic growth, and this is mainly realised from the medium to long-term perspective for most economies. In this aspect, financial system expansion would necessitate global investors maximizing their profits through more productive investments. However, China exhibits the least dependency concerning finance growth nexus, followed by India. Policymakers and other regulators of China and India should take a critical look at this outcome to ensure that their financial sector activities proceed with caution. This is because, countries with less developed financial systems have a higher likelihood of experiencing market failure issues as a result of incomplete competition and information asymmetry [89].

Evidence from the wavelet multiple correlations analysis indicates that the RFSI is the first mover at most time scales for the BRICS economies. This is followed by GEPU which either leads or lags for most scales, especially for South Africa. Consequently, we found that GDP lags for South Africa which is vulnerable to direct shocks from the GEPU. The lagging property for GDP in South Africa signifies that it is the last variable to respond to shocks, but the impact will most likely be significant. However, GDP has the potential to lead/lag the variables for Russia. The impact of GEPU on RFSI and GDP is worst for South Africa in about four cases in the medium-, and long-terms. This signifies that South Africa’s financial markets and economic growth are vulnerable to external uncertainty shocks. However, GEPU lags for India, but has a potential to lead or lag for Russia and China in the medium-term. This outcome contributes to the findings of Osei et al. [106] that EPU from China responds quickly to a rise in value of other ASIAN countries’ EPU indices, which may require possible policy uncertainty synergies and spillovers among the Asian economies. Despite the result of this study, Carré and L’œillet [107] in their review indicated that the nexus between finance-growth nexus is inconclusive.

We confirmed from the findings that the COVID-19 pandemic had an adverse impact on the contribution of RFSI to GDP when the pre-COVID-19 pandemic analysis was conducted for the multiple wavelet. The pre-COVID-19 pandemic sample analysis was needed for only multiple wavelet technique because its econometric properties allow us to assess only frequency domain. The bi-wavelet and partial-wavelet techniques which present the analysis for time-frequency domain also indicated that the COVID-19 pandemic had a significant impact on the BRICS economies’ RFSI, GDP and GEPU. Consequently, it was clear that the GEPU had the less tendency to drive the RFSI and GDP for all economies in the pre-COVID-19 pandemic sample relative to the full sample scenario. This indicates that the COVID-19 pandemic has distorted most economic activities and heightened the level of uncertainties rendering effective assets allocation and portfolio diversification as part of the optimum choice for investors.

The study offers an important empirical contribution to prior literature on finance-growth nexus, and also has practical consequences. First, we demonstrate that the RFSI of these emerging markets can be used to measure the performance of the financial sector. This enables a decoupling of the growth in the financial sector from the other sectors of their economies for a better assessment of the finance-growth nexus. Second, the outcomes from the study reveal the importance of the financial market to global investors by maximising their investment potentials to increase financial market integration among emerging economies. These developed relationship will enable economic growth to cushion the development of the financial sector against adverse external uncertainty shocks on financial markets. That is, from the demand-following perspective, economic growth would tend to increase the demand for financial services leading to financial sector development. It is recommended that policymakers, investors and researchers across the globe should incessantly observe the relationship between financial sector development and economic growth across time while considering adverse shocks from global economic policy uncertainty. To reveal hidden relationships, further studies may apply quantile regression [108] or transfer entropy [109] to examine the finance-growth nexus.

## Supporting information

S1 File (ZIP)
